# A Framework for Cybersecurity Requirements Management in the Automotive Domain

**DOI:** 10.3390/s23104979

**Published:** 2023-05-22

**Authors:** Feng Luo, Yifan Jiang, Jiajia Wang, Zhihao Li, Xiaoxian Zhang

**Affiliations:** 1 School of Automotive Studies, Tongji University, Shanghai 201804, China; luo_feng@tongji.edu.cn (F.L.); 1911047@tongji.edu.cn (J.W.); 2011451@tongji.edu.cn (Z.L.); 2 iSOFT Infrastructure Software Co., Ltd., Shanghai 200125, China; alex.zhang@i-soft.com.cn

**Keywords:** security requirements engineering, formal methods, threat analysis and risk assessment, security specification

## Abstract

The rapid development of intelligent connected vehicles has increased the attack surface of vehicles and made the complexity of vehicle systems unprecedented. Original equipment manufacturers (OEMs) need to accurately represent and identify threats and match corresponding security requirements. Meanwhile, the fast iteration cycle of modern vehicles requires development engineers to quickly obtain cybersecurity requirements for new features in their developed systems in order to develop system code that meets cybersecurity requirements. However, existing threat identification and cybersecurity requirement methods in the automotive domain cannot accurately describe and identify threats for a new feature while also quickly matching appropriate cybersecurity requirements. This article proposes a cybersecurity requirements management system (CRMS) framework to assist OEM security experts in conducting comprehensive automated threat analysis and risk assessment and to help development engineers identify security requirements prior to software development. The proposed CRMS framework enables development engineers to quickly model their systems using the UML-based (i.e., capable of describing systems using UML) Eclipse Modeling Framework and security experts to integrate their security experience into a threat library and security requirement library expressed in Alloy formal language. In order to ensure accurate matching between the two, a middleware communication framework called the component channel messaging and interface (CCMI) framework, specifically designed for the automotive domain, is proposed. The CCMI communication framework enables the fast model of development engineers to match with the formal model of security experts for threat and security requirement matching, achieving accurate and automated threat and risk identification and security requirement matching. To validate our work, we conducted experiments on the proposed framework and compared the results with the HEAVENS approach. The results showed that the proposed framework is superior in terms of threat detection rates and coverage rates of security requirements. Moreover, it also saves analysis time for large and complex systems, and the cost-saving effect becomes more pronounced with increasing system complexity.

## 1. Introduction

The complexity of automotive systems is rapidly increasing due to the incorporation of advanced features and technologies. The automotive industrial sectors are currently confronted with massive difficulties originating from managing the increasing complexity of systems [1]. As more devices become automated, the volume of embedded software is increasing at 10 to 20 percent per year, depending on the domain [2]. As a result, the amount of software code required to operate these systems is also rapidly increasing. This increase in software code introduces a multitude of security risks that must be addressed to ensure the safety and reliability of the vehicle. These risks include potential vulnerabilities that could be exploited by attackers as well as unintended consequences resulting from complex interactions between system components. Xiong et al. [3] collected and analyzed vulnerabilities and software weaknesses in vehicles from the National Vulnerability Database (NVD). The three most common types of vulnerabilities identified were protection mechanism failure, buffer errors, and information disclosure. Among these, the most frequent type of vulnerability was protection mechanism failure, which refers to instances where the vehicle fails to use or improperly uses a protection mechanism that can provide adequate defense against targeted attacks directed at the vehicle. To mitigate these risks, it is essential to accurately and rapidly identify system risks and match them to corresponding security requirements. This requires a comprehensive and systematic approach to risk identification and mitigation that takes into account the entire system architecture and all potential attack vectors. By leveraging advanced techniques such as threat modeling and formal verification, it is possible to gain a deeper understanding of the security risks associated with automotive systems and develop effective strategies for mitigating those risks.

In the current automotive industry, where there is a highly competitive environment and rapid iteration of new functions is required, it is necessary for software engineers to develop engineering code that meets a series of strict security requirements in order to accurately describe and identify threats and risks in complex systems. This requires software engineers to possess both professional coding abilities and a certain level of expertise in information security. However, it is currently unrealistic or significantly increases the human resources costs of original equipment manufacturers (OEMs). Therefore, a cybersecurity requirements management framework tailored to the automotive industry is needed, which can meet the complex threat and risk descriptions of security experts for high-complexity systems to achieve accurate risk identification and can also help software engineers obtain corresponding security requirements before code development to assist them in completing agile development.

Many studies have proposed their own solutions to address this issue. Predrag Filipovikj et al. [4] proposed a pattern-based approach to formally describe security requirements, seeking successful industrial applications of security requirements engineering. The EAST-ADL (i.e., Electronics Architecture and Software Technology—Architecture Description Language) framework language was proposed in [5] to describe the automotive domain in accordance with the ISO 26262 standard. In article [6], a flexible model-driven engineering-based architecture named Flex-eWare was proposed for designing and implementing embedded distributed systems, allowing for relatively complete modeling of embedded distributed systems. However, some of these solutions fail to balance the precision of system threat modeling with the ease of use for software engineers, while others are not specifically designed for the automotive domain and are unable to perform targeted modeling and analysis of automotive domain communication protocols, among other issues.

In this paper, we have proposed a cybersecurity requirements management system (CRMS) as a security requirement analysis framework applied to the automotive industry. The ultimate goal of the CRMS is to assist security experts in OEMs in conducting comprehensive automated threat analysis and risk assessment of the entire system, as well as to help system development engineers obtain the necessary security requirements before implementing software development. In article [7], a complete review of threat analysis and risk assessment methods for automotive systems is presented. This review mentions that the threat analysis and risk assessment methods in the automotive field are mainly divided into two categories: formula-based methods and model-based methods. Model-based methods mainly include Unified Modeling Language (UML) and Systems Modeling Language (SysML) methods, which have the advantage of being able to intuitively display components, communication channels, and attributes in the system, but the description of system details is not sufficiently detailed. Formula-based methods mainly include first-order logic-based methods and Markov decision processes (MDP), which have the advantage of being able to describe the system in detail at a finer granularity, but their learning cost is higher.

Meanwhile, in order to reduce the communication difficulty and time cost between system development engineers and security experts, our proposed cybersecurity requirements management system needs to provide secure interfaces that can adapt to the professional skills of both parties. System development engineers prefer to use graphical and concise descriptions of the system through the UML-based method in the model-based approach, requiring visual views that are easy to understand and use. Security experts, on the other hand, need to model the system more accurately and rigorously for security, enabling them to identify threats more precisely. It is easy to see that the advantages and disadvantages of the two methods of threat analysis and risk assessment described in article [7] match the requirements and abilities of system development engineers and security experts. Therefore, our proposed approach is to allow system development engineers to use the UML-based method in the model-based approach, while security experts use the first-order logic based method in the formula-based approach based on the STRIDE (i.e., Spoofing, Tampering, Repudiation, Information disclosure, Denial of service, and Elevation of privilege) threat analysis model to model the system, and then translate and match between the two to achieve the goal of helping security experts conduct comprehensive automated threat analysis and risk assessment of the entire system and helping software development engineers obtain security requirements that should be added to the system before implementing software development.

Based on the above considerations, a framework for cybersecurity requirements management in the automotive domain called CRMS is proposed in this paper. The contributions of this paper can be summarized as follows:

1. An accurate and fast threat analysis and security requirements system called CRMS, suitable for complex large-scale automotive systems, is proposed;
2. A secure communication framework for the automotive domain, named the component channel messaging and interface (CCMI) framework, which can serve as a middleware for converting formal and informal representation methods, has been proposed;
3. The proposed approach is applied to an autonomous emergency braking (AEB) system. Its risk detection rate, coverage rates of security requirements, and analysis time were evaluated and compared with the HEAVENS method.

The rest of this paper is organized as follows: Section 2 introduces the related work of security requirement management methods. Section 3 introduces the concepts of first-order logic and the formal tool Alloy. Section 4 presents the proposed framework for cybersecurity requirements management in the automotive domain. Section 5 presents an illustration for applying the proposed CRMS framework to the autonomous emergency braking system. Finally, Section 6 concludes this paper.

## 2. Related Work

There are now many studies on threat risk analysis and security requirements engineering. The methods of threat analysis and risk assessment are mainly divided into formula-based and model-based methods. Formula-based methods can be divided into asset-based methods, vulnerability-based methods, and attacker-based methods. Asset-based methods, such as the EVITA method (i.e., e-safety vehicle intrusion protected applications), evaluate the risk level of each asset in the system by performing an attack assessment and then assessing the potential level of risk that an attack could cause [8]. However, such methods only provide evaluation techniques and do not offer a complete evaluation process. The HEAVENS (i.e., healing vulnerabilities to enhance software security and safety) method complements this limitation and is a suitable assessment method for evaluating information security risks in automotive electronic and electrical systems [9]. The HEAVENS method first requires users to identify their security attributes and security goals and provide corresponding evaluation objects. Then, the Microsoft STRIDE threat analysis model is used for threat analysis. STRIDE associates threats with security attributes, and each type of STRIDE threat is statically mapped to a set of security attributes, including spoofing, tampering, repudiation, information disclosure, denial of service, and elevation of privilege, corresponding to authentication, integrity, non-repudiation, confidentiality, availability, and authority, respectively. Threats and evaluation objects are then categorized by considering both the threat level (TL) and impact level (IL) dimensions, leading to a security level classification. Finally, the four dimensions of threat, evaluation object, security attribute, and security level are integrated to form security requirements. Multi-metric approaches, such as SHIELD [1,10], evaluate the system’s level of security, privacy, and dependability by identifying multiple system configurations that meet established requirements. The SGM (i.e., security guide-word method) method, on the other hand, simplifies the identification of information assets and protection objectives, making it easy for non-security engineers to use [11]. TVRA (i.e., threat vulnerability and risk analysis) defines the risk level of a system based on the probability of an attack occurring and the impact it could have on the system and can output a quantitative measure of system asset risk and a detailed set of security measures to minimize risk [12]. Macher et al. [13] proposed a method called SAHARA (i.e., security-aware hazard analysis and risk assessment), which incorporates the STRIDE threat model. Vulnerability-based methods, such as FMVEA (i.e., failure mode vulnerabilities and effects analysis) [14], analyze a vulnerability’s potential to cause larger vulnerabilities or failures in the system. CHASSIS (i.e., combined harm assessment of safety and security for information systems) defines functionality, safety, and security requirements in two steps [15]. ANP (i.e., analytical network process) is a matrix approach that can effectively consider dependencies and conflicts between attributes for joint evaluation [16]. Attacker-based methods, such as SARA (i.e., security automotive risk analysis) [17], conduct threat analysis and risk assessment based on the knowledge level of possible attackers, attack paths, attack motivations, and resources possessed. SAM (i.e., security abstraction model) [18] combines safety management and model-based system engineering through an abstract description of automotive security modeling. The Bayesian Stackelberg game methodology models the attack and defense processes as a network security Stackelberg game [19].

Model-based methods can be divided into graph-based methods and tree-based methods. Graph-based methods use nodes and directional edges to represent the system’s structure. The STRIDE model, which consists of six categories, namely spoofing, tampering, repudiation, information disclosure, denial of service, and elevation of privilege, has been widely used for threat analysis in software engineering [20]. PASTA (i.e., process for attack simulation and threat analysis) [21], a seven-stage threat analysis method, complements the STRIDE method. Markov chain methods can perform quantitative threat analysis, providing a more intuitive and convincing result for the entire system [22,23,24,25,26]. The GTS (i.e., graph transformation system) method is a formal method of transforming the system structure graph, which can easily and quickly realize the conversion between the overall architecture and the module architecture [27]. UMLsec (i.e., Unified Modeling Language Security), a UML extension, has been used for model-based security engineering in distributed information systems [28,29]. Shah et al. [30] present a method of automatically creating a model transformation based on the original UML2Alloy transformation. Attack tree analysis, a tree-based method, uses a tree structure to represent the hierarchical relationships between nodes and has been widely used for threat analysis. The RISKEE (i.e., risk-tree) [31] method calculates risk through forward propagation of frequencies and backward propagation of risk using the RISKEE propagation algorithm. Moreover, many studies have proposed improvements and extensions to existing methods, such as STPA-Sec (i.e., systems theoretic process analysis—security) [32] and STPA-SafeSec (i.e., systems theoretic process analysis—safety and security) [33]. The advantages and disadvantages of different relevant methods have been summarized in Table 1.

Furthermore, Ansari et al. [34] proposed a methodology named STORE for eliciting security requirements based on security threat analysis. This methodology includes the identification of four key points for effective security attack analysis: the point of attack, the point of belief, the point of conjecture, and the point of dependency. In addition, the article [35] proposes a practical repository model for reusable security requirements that is user-friendly and understandable even for non-security experts. Mouratidis et al. [36] presented a novel security modeling language and a set of original analysis techniques for capturing and analyzing security requirements for cloud computing environments. However, these approaches have not been adapted to the protocols used in the automotive industry and may not be directly applicable to security engineering in the automotive field. Bouskela et al. [37] presented an integrated solution for formally defining system requirements and automating their verification through simulation. Kumar et al. [38] proposed a supervised text categorization approach for automatically extracting and classifying non-functional requirements. However, these methods require a high level of expertise and may be challenging for software developers with limited knowledge of cybersecurity. Therefore, there is an urgent need for a network security requirement analysis and management framework in the automotive industry that can address all of these pain points.

## 3. Preliminaries of Proposed Approach

### 3.1. First-Order Logic

First-order logic, also known as first-order predicate calculus, is a branch of mathematical logic that deals with logical systems based on objects, properties, and relations. In first-order logic, there is a set of objects, and these objects can be assigned properties and related to each other. It can be used to describe complex propositions in natural language and provide tools and methods for analyzing and proving these propositions.

The language of first-order logic can be represented by a set of symbols, including constants, variables, predicates, functions, and logical symbols. Constants are symbols that represent fixed objects; variables are symbols that represent any object. Predicates represent relations or properties, and functions map a set of objects to another set of objects. Logical symbols include negation, conjunction, disjunction, implication, biconditional, and quantifiers.

First-order logic has the following characteristics:

- Precision: First-order logic is a precise mathematical language that can describe complex problems and various relationships clearly and accurately;
- Completeness: First-order logic is a complete language that can describe any countable thing or relationship;
- Natural expression: First-order logic has a natural expression form that can express and understand complex problems and relationships in a human-readable form;
- Computability: First-order logic has computability and can perform automatic reasoning and proof through computers;
- Scalability: First-order logic has high scalability and can handle new problems and domains by adding new symbols and rules;
- Wide application: First-order logic is widely used in formal language, formal proof, and formal methods, especially in computer science, artificial intelligence, and human-computer interaction.

By using first-order logic, the accuracy and precision of problems can be improved, the difficulty of reasoning and proof can be reduced, the efficiency of computer automatic reasoning can be improved, and more reliable and effective solutions can be provided in various fields.

### 3.2. Alloy

Alloy is a formal modeling language and analyzer that is widely used in industry and academia to model and analyze complex systems, particularly in the context of safety and security-critical applications. Alloy’s main advantage over traditional modeling and analysis tools is its ability to integrate high-level, declarative modeling with formal, precise reasoning using first-order logic.

At its core, Alloy is a language for describing abstract models of systems in a way that is both intuitive and precise. The language is based on a subset of predicate calculus, which is a formal language for expressing logical statements about sets and their elements. In Alloy, models are expressed as collections of sets and predicates, which describe the relationships and constraints that govern the behavior of the system.

One of the main advantages of Alloy is its ability to automatically generate and explore the space of possible models that satisfy a given set of constraints. The Alloy analyzer, which is a tool for analyzing Alloy models, uses a SAT solver to explore the space of possible models up to a certain depth or bound and returns a counterexample if the constraints are not satisfied. The analyzer can also generate visualizations of the models and counterexamples, which can help in debugging and understanding the model.

The integration of Alloy with first-order logic provides several advantages over traditional modeling and analysis tools. First, it allows the user to express complex, abstract models in a high-level, declarative way while still being able to reason about their properties using the precise, formal language of first-order logic. This makes it easier for the user to understand and reason about the model, as well as to communicate it to others. Second, it allows the user to take advantage of the powerful tools and techniques developed for first-order logic, such as automated theorem proving and deductive reasoning, to verify the properties of the model.

Alloy’s ability to generate counterexamples is also a powerful tool for detecting design flaws and potential security vulnerabilities in complex systems. By automatically exploring the space of possible models and returning counterexamples when the constraints are not satisfied, Alloy provides a systematic way to check the correctness of the model and identify potential problems. This makes it a valuable tool for modeling and analyzing safety and security-critical systems, where even small design flaws or errors can have catastrophic consequences.

Overall, Alloy is a powerful tool for modeling and analyzing complex systems, particularly in the context of safety and security-critical applications. Its integration with first-order logic provides a formal, precise language for reasoning about the model, while its high-level, declarative syntax makes it easier to specify and understand complex models. The Alloy analyzer provides an automatic solver that greatly reduces the need for the user to manually explore the search space, as well as a counterexample generator and a graphical visualization tool that help in debugging and understanding the model.

Alloy’s ability to integrate high-level modeling with formal, precise reasoning using first-order logic is a key advantage that sets it apart from traditional modeling and analysis tools. By providing a systematic way to check the correctness of the model and identify potential problems, Alloy is a valuable tool for modeling and analyzing safety and security-critical systems.

## 4. Proposed Approach

### 4.1. Cybersecurity Requirements Management System (CRMS)

**Figure 1:**
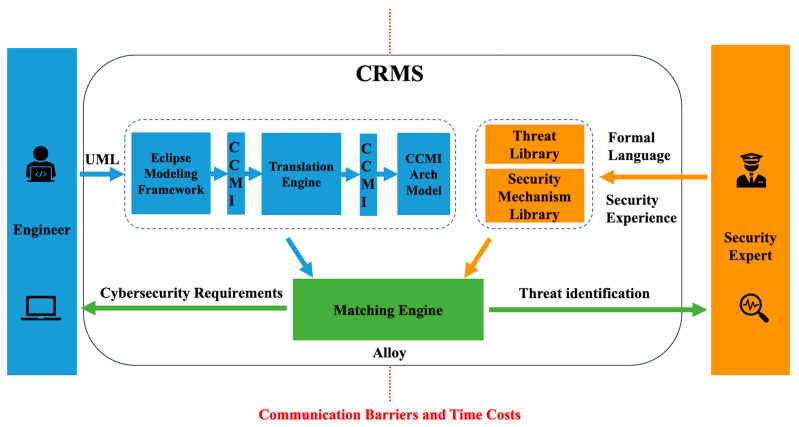
Cybersecurity requirements management system framework.

The cybersecurity requirements management system aims to reduce communication difficulties and time costs between system development engineers and security experts by providing security interfaces that adapt to their functional and security-related professional skill levels. System development engineers seek a graphical, clear, and concise description of the system through model-based methods, requiring visual views for easier understanding and use. Security experts require more precise and rigorous security modeling of the system to identify threats more accurately. Therefore, our proposed approach is to allow system development engineers to use the UML-based method of model-based methods and security experts to use the first-order logic-based method of formula-based methods. These methods are translated and matched to enable security experts to perform automated, comprehensive threat analysis and risk assessment of the entire system and to help software development engineers obtain security requirements that should be added to the system’s functionality before implementing software development. This complete process and mechanism are called CRMS, with its architecture diagram shown in Figure 1.

**Figure 2:**
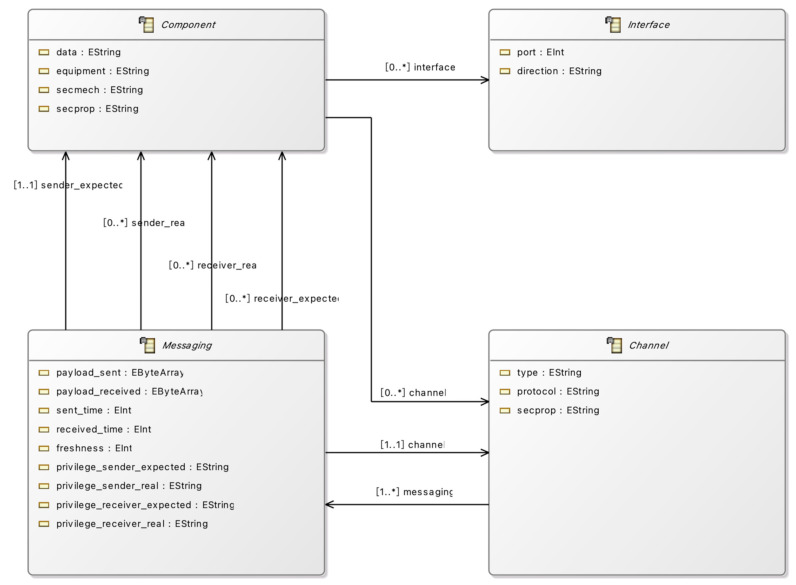
UML diagram of CCMI communication framework represented in EMF. *: map to multiple targets and is correct and accurate.

System development engineers use UML to comprehensively model the vehicle system in Eclipse and convert the UML model into Java code using the Eclipse Modeling Framework (EMF). UML model diagrams enable system development engineers to describe the entire vehicle system’s components and communication channels. However, the issue of how UML models can be matched with threat libraries and security mechanism libraries built using formal languages by security experts remains unresolved. To solve this issue, we introduce the concept of middleware to facilitate this conversion process. We have constructed an intelligent CCMI communication model framework exclusively for intelligent connected vehicles. By inputting the Java system model generated by EMF into our CCMI communication model framework, different standardized instantiated models that comply with the CCMI communication model in various application scenarios can be generated. This enables matching with the threat library and security mechanism library built by security experts using formal languages. We will provide a detailed introduction to the CCMI communication model framework in Section 4.2, and the CCMI communication framework expressed in UML diagrams in EMF is shown in Figure 2. The mapping table between the Java system model generated by EMF and the alloy language used in the CCMI system framework is simple, as shown in Table 2. Therefore, the CCMI communication model framework enables the coupling relationship between UML system framework expression and formal verification, greatly reducing conversion difficulty and saving conversion time.

### 4.2. CCMI Secure Communication Framework

The CCMI secure communication framework consists of four parts, namely component, channel, messaging, and interface, represented in UML diagrams as shown in Figure 2. Component refers to the communication entity, while interface represents the communication interface used by the communication entity. Channel is primarily used to characterize the medium used for communication, such as CAN FD or Ethernet, and may include a protocol attribute to represent the protocol used for communication, such as SOME/IP or MQTT. Messaging refers to the act or process of exchanging information, encompassing a range of activities such as sending, receiving, tampering, and sniffing messages, as well as managing the security properties and related behaviors of messages during the message passing process. On the other hand, a message is a unit of communication that is sent or received between two or more parties and takes various forms such as text, voice, video, or data. It is often used to convey information, instructions, or requests.

In essence, messaging is a broader term that encompasses the entirety of the communication process, while message refers to a discrete unit of information transmitted as part of that process. As a result, messaging can provide a more comprehensive and accurate representation of the complex and heterogeneous communication between ECUs in intelligently connected vehicles. Below is a detailed description of the CCMI secure communication framework:

**Definition** **1.**  *CCMI consists of a quadruple*

(1) $$CCMI=\langle C,\psi ,M,J\rangle$$

where

- C denotes Component, which is a finite set of modules, where each element represents a module in the system;
- ψ denotes Channel, which is a finite set of communication channels, where each element represents a communication channel that messages pass through to be transmitted between different modules;
- M denotes Messaging, which is an abstract concept of message passing, describing the dynamic process of messages and their security properties such as sender and receiver modules, communication channels used, message payloads, and message freshness;
- J denotes a finite set of interfaces, where all messages in the system must communicate between different modules’ interfaces.

**Definition** **2.**  C *consists of a sextuple*

(2) $$C≜\left\{C|C=\langle J,\psi ,Data,Equip,R,S\rangle \right\}$$

where

- J denotes the set of all interfaces connected to C;
- ψ denotes the set of all communication channels connected to C, through which messages are transmitted between different modules;
- Data denotes the set of all data stored in this module, including normal data, sensitive data, and personal data;
- Equip denotes the set of all security devices deployed in this module, such as HSM;
- R denotes the set of all security mechanisms deployed in this module;
- S denotes the set of all security attributes of this module.

**Definition** **3.**  ϕ *is composed of a quadruple*

(3) $$\phi ≜\left\{\phi |\phi =\langle Type,P,H,M\rangle \right\}$$

where

1. Type denotes a set of types for the given ψ, including Ethernet, CAN FD, LVDS, etc.
2. P denotes a set of communication protocols used for message exchange over the given ψ. The protocols are organized into 5 layers based on the TCP/IP model: application layer, transport layer, network layer, data link layer, and physical layer. The TCP/IP model is a widely used communication protocol layer model in intelligent connected vehicles. For example, the SOMEIP protocol is an application-layer protocol. By fully including the 5-layer protocols of TCP/IP, we can perform a fine-grained security attribute analysis of the entire vehicle network and thus conduct a comprehensive security assessment of the entire vehicle;
3. H denotes a set of all security attributes of the communication channel;
4. M denotes a set of all messages transmitted over the communication channel.

**Definition** **4.**  (4) $$M≜\left\{M|M=\langle \bar{S},\tilde{S},\bar{R},\tilde{R},\psi ,\bar{P},\tilde{P},\alpha ,\beta ,\delta ,\bar{PS},\tilde{PS},\bar{PR},\tilde{PR}\rangle \right\}$$

where

- $\bar{S}$ denotes the predetermined sending module of the message;
- $\tilde{S}$ denotes the actual sending module of the message;
- $\bar{R}$ denotes the predetermined set of receiving modules for the message;
- $\tilde{R}$ denotes the actual set of receiving modules for the message;
- ϕ denotes the set of communication channels that carry the message;
- $\bar{P}$ denotes the expected data payload of the message when sent by the sending module;
- $\tilde{P}$ denotes the actual data payload of the message when received by the receiving module;
- α denotes the actual time when the message is sent;
- β denotes the actual time when the message is received;
- δ denotes the freshness value of the message;
- $\bar{PS}$ denotes the expected set of permissions for the message by the sending module;
- $\tilde{PS}$ denotes the actual set of permissions for the message by the sending module;
- $\bar{PR}$ denotes the expected set of permissions for the message by the receiving module;
- $\tilde{PR}$ denotes the actual set of permissions for the message by the receiving module.

**Definition** **5.**  (5) $$J≜\left\{J|J=\langle N,D\rangle \right\}$$

where

- N is the port number of the interface, which can be set to a default value if the connected protocol does not define the concept of port numbers;
- D is the message transmission direction of the interface, including input, output, and duplex.

### 4.3. STRIDE Threat Model Specification

STRIDE is a threat model developed by Microsoft to identify and classify different types of security threats to software systems. It consists of six categories of threats: spoofing, tampering, repudiation, information disclosure, denial of service, and elevation of privilege.

Spoofing threats involve a malicious actor pretending to be someone or something else in order to gain access to a system or perform actions they should not be able to perform. Tampering threats involve the unauthorized modification of data or code within a system. Repudiation threats involve a malicious actor denying that they performed a particular action within a system. Information disclosure threats involve the unauthorized exposure or release of sensitive information. Denial-of-service threats involve the disruption or blocking of legitimate access to a system or resource. Elevation of privilege threats involve a malicious actor gaining increased access or permissions within a system.

By considering these six categories of threats, software developers and security professionals can develop security measures to detect and prevent potential attacks. This may include implementing access controls, encryption, auditing, and monitoring systems.

#### 4.3.1. Spoofing

The spoofing threat refers to the malicious act of forging the identity of a legitimate message sender by a malicious node so that the message receiver perceives it as originating from a legitimate node and processes its content accordingly. Since the message is sent by a malicious node, it is likely to contain a malicious payload that can cause damage to the system if executed by the receiver.

For $M\in \phi \left(M\right)$ means that the message M is transmitted over the communication channel ϕ. Detection of spoofing threats can be realized by:

(6) $$\forall m\epsilon M,\phi \epsilon \psi ,C_{S}\epsilon C,c_{s}\epsilon C_{S}|P(m,c_{s})⋀Q\left(m,c_{s}\right)⟹\neg Spoofing\left(\phi \right)$$

where M denotes the set of messages, ψ denotes the set of all communication channels, and $C_{S}$ denotes the set of all possible senders. $P\left(m,c_{s}\right)$ indicates that message m is designed to be sent by module $c_{s}$; $Q\left(m,c_{s}\right)$ indicates that the actual sender of message m is module $c_{s}$. If both conditions are satisfied, it can be concluded that all messages M transmitted through the communication channel ϕ are not subject to spoofing threats.

#### 4.3.2. Tampering

Tampering involves modifying the data payload of a message to alter its intended meaning or behavior. Attackers may use various techniques, such as data injection and code injection, to modify the behavior of a system or application.

For $M\in \phi \left(M\right)$ means that the message M is transmitted over the communication channel ϕ. Detection of tampering threats can be realized by:

(7) $$\forall m\epsilon M,\phi \epsilon \psi |m(\bar{P})=m(\tilde{P})⟹\neg Tampering\left(\phi \right)$$

where the message set is denoted by M, ψ denotes the set of all communication channels. $m(\bar{P})$ denotes the expected data payload of message m when sent from the sending module, while $m(\tilde{P})$ denotes the actual data payload of message m received by the receiving module. If these two values are equal, it can be determined that all messages M transmitted through the communication channel ϕ are not subject to tampering threats.

#### 4.3.3. Repudiation

Repudiation refers to the denial by a sender or receiver of having sent or received a message, creating uncertainty and confusion about the authenticity of the message. Attackers can exploit vulnerabilities in the system to forge or manipulate message headers or content to hide their actions.

For $M\in \phi \left(M\right)$ means that the message M is transmitted over the communication channel ϕ. Detection of repudiation threats can be realized by:

(8) $$\forall m\epsilon M,\phi \epsilon \psi ,C_{S},C_{R}\epsilon C,c_{s}\epsilon C_{S}|P(m,c_{s})⋀Q\left(m,c_{s}\right)⋀U(m,C_{R})⋀V\left(m,C_{R}\right)⟹\neg Repudiation\left(\phi \right)$$

where M represents a set of messages, ψ represents a set of all communication channels, $C_{S}$ represents a set of all possible senders, and $C_{R}$ represents a set of all receivers. $P\left(m,c_{s}\right)$ represents the message m being designed to be sent by module $c_{s}$. $Q\left(m,c_{s}\right)$ represents the actual sender of message m being module $c_{s}$. $U\left(m,C_{R}\right)$ represents the message m being designed to be received by a set of modules $C_{R}$. $V\left(m,C_{R}\right)$ represents the actual receiver of message m being a set of modules $C_{R}$. If all these conditions are met, then it can be determined that all messages in the set M transmitted through the communication channel ϕ will not be subjected to the repudiation threat.

#### 4.3.4. Information Disclosure

Information disclosure occurs when an attacker gains access to confidential or sensitive data that is not intended for them. This can occur through various means, such as hacking, social engineering, or exploiting vulnerabilities in the system.

For $M\in \phi \left(M\right)$ means that the message M is transmitted over the communication channel ϕ. Detection of information disclosure threats can be realized by:

(9) $$\forall m\epsilon M,\phi \epsilon \psi ,C_{R}\epsilon C|U(m,C_{R})⋀V\left(m,C_{R}\right)⟹\neg InformationDisclosure\left(\phi \right)$$

where M represents the set of messages, ψ represents the set of all communication channels, $C_{R}$ represents the set of all recipients. $U\left(m,C_{R}\right)$ indicates that message m is designed to be received by a set of modules $C_{R}$. $V\left(m,C_{R}\right)$ indicates the actual recipient set of message m is $C_{R}$. If these conditions are met, it can be concluded that all messages M transmitted through communication channel ϕ will not be subject to information disclosure threats.

#### 4.3.5. Denial of Service

Denial of service (DoS) attacks aim to disrupt the normal functioning of a system by overwhelming it with a flood of requests, rendering it unresponsive or unavailable, ultimately preventing the successful delivery of messages within their validity period. Attackers may use various techniques, such as flooding, amplification, or distributed attacks, to generate massive traffic or requests for a target system or network.

For $M\in \phi \left(M\right)$ means that the message M is transmitted over the communication channel ϕ. Detection of Denial of Service threats can be realized by:

(10) $$\forall m\epsilon M,\phi \epsilon \psi |m(\beta )<(m(\alpha )+m(\delta ))⟹\neg DenialofService\left(\phi \right)$$

where M denotes a set of messages, ψ represents the set of all communication channels. α represents the actual sending time of a message, β represents its actual receiving time, and δ represents the freshness value of the message. If all the messages in M that are transmitted over the communication channel ϕ can be delivered to the recipients before their freshness value expires, then it can be concluded that there is no denial of service threat on the communication channel ϕ.

#### 4.3.6. Elevation of Privilege

Elevation of privilege attacks involve the unauthorized escalation of privileges to gain access to resources and information that are not normally accessible to a user. This can be accomplished by exploiting vulnerabilities in the system, taking advantage of weak authentication, or using social engineering tactics to deceive users into granting access.

For $M\in \phi \left(M\right)$ means that the message M is transmitted over the communication channel ϕ. Detection of elevation of privilege threats can be realized by:

(11) $$\begin{array}{c} \forall m\epsilon M,\phi \epsilon \psi ,C_{S},C_{R}\epsilon C,\\ \;\;\;\;\;\;c_{s}\epsilon C_{S}|I(m,c_{s},\overset{-}{PS})⋀J\left(m,c_{s},\bar{PS}\right)⋀M(m,C_{R},\bar{PR})⋀N\left(m,C_{R},\bar{PR}\right)\\ \;\;\;\;\;\;⟹\neg ElevationofPrivilege\left(\phi \right) \end{array}$$

where M represents a set of messages, ψ represents a set of all communication channels, $C_{S}$ represents a set of all possible senders, $C_{R}$ represents a set of all receivers. $I(m,c_{s},\bar{PS})$ represents the expected set of permissions for the sending module $c_{s}$ of the message m is $\bar{PS}$. $J(m,c_{s},\bar{PS})$ represents the actual set of permissions for the sending module $c_{s}$ of the message m, which is the same as the expected set of permissions $\bar{PS}$. $M(m,C_{R},\bar{PR})$ represents the expected set of permissions for the receiving module set $C_{R}$ of the message m is $\bar{PR}$. $N(m,C_{R},\bar{PR})$ represents the actual set of permissions for the receiving module set $C_{R}$ of the message m, which is the same as the expected set of permissions $\bar{PR}$. If all of these conditions are met, it can be determined that all messages M transmitted over communication channel ϕ will not be subject to elevation of privilege threats.

After defining the formal logic of six threats, we can combine these formalized logics with the security attributes of components or channels in our proposed CCMI model. The integrated security attribute checking logic will be integrated into our formalized threat library (which will be introduced in Section 5). After describing the actual system using our proposed framework, we can use our formalized threat library to check the security attributes of different assets in the actual system to see if they match the formalized logic of the above threats. If a match is found, it indicates that the asset has a corresponding threat. Otherwise, it indicates that the corresponding security attributes of the asset are protected and there is no matching risk of this kind.

## 5. Illustration

**Figure 3:**
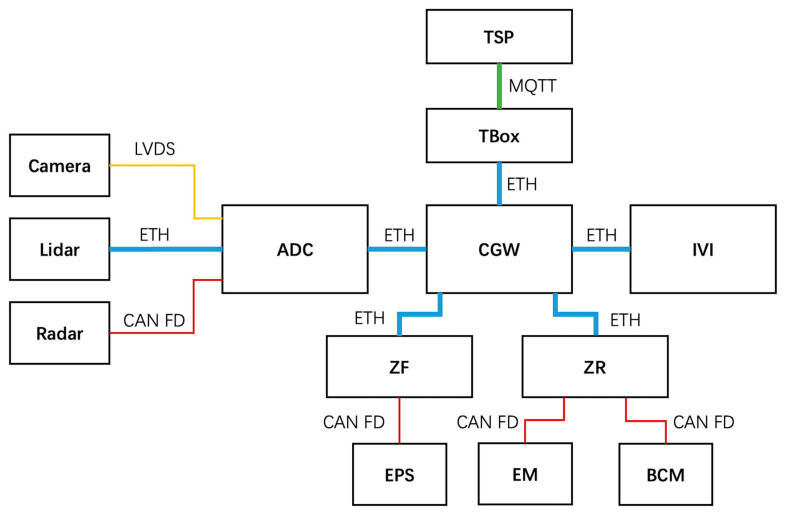
Autonomous emergency braking architecture diagram.

Autonomous emergency braking (AEB) is an advanced driver assistance system (ADAS) that automatically applies the brakes of a vehicle to prevent or mitigate a collision. AEB is a key feature of intelligent connected vehicles (ICVs), as it can significantly reduce the risk of crashes and save lives. With the increasing adoption of ICVs, AEB has become a critical technology for enhancing passenger safety. Its main architecture is shown in Figure 3. AEB systems use a variety of sensors, such as radar, lidar, and cameras, to detect potential collisions with other vehicles, pedestrians, or obstacles. When a collision is imminent, the system can automatically apply the brakes to avoid or reduce the severity of the impact. AEB is particularly effective at preventing or mitigating rear-end collisions, which are among the most common types of crashes.

Despite its potential to improve passenger safety, AEB also poses significant information security threats. The sensors used in AEB systems are vulnerable to a variety of attacks, including jamming, spoofing, and replay attacks. An attacker could use these attacks to manipulate the sensor data and cause the AEB system to apply the brakes unnecessarily or not apply them when needed. This could lead to accidents or create opportunities for other types of attacks. Furthermore, the AEB system is also vulnerable to software and firmware attacks, which could be used to exploit vulnerabilities in the system and compromise its security. An attacker could gain unauthorized access to the system and modify its behavior, potentially causing harm to passengers or other road users.

To address these threats, it is essential to conduct a thorough security analysis of AEB systems and implement appropriate security measures. This could include the use of secure communication protocols between the various components of the AEB system, the integration of intrusion detection and prevention systems, and the adoption of secure software development practices. Additionally, regular security testing and maintenance should be conducted to ensure that the AEB system remains secure and effective.

In our AEB system, vehicles are able to detect the distance and movement speed of different obstacles in front of them through cameras, lidar, and radar and transmit the monitored data to the ADAS domain controller through different communication channels after preprocessing the data internally. A camera is a sensor that captures images of the environment in front of the vehicle and is typically used for object detection, recognition, and tracking. The images captured by the camera are usually transmitted to the ADAS domain controller via LVDS. Lidar, on the other hand, uses laser beams to measure distances to objects in the environment and is useful for generating a 3D map of the environment around the vehicle. Due to the large amount of data generated by the Lidar, Ethernet is used for data transmission between the Lidar and the ADAS domain controller. Radar is a sensor that uses radio waves to detect the presence of objects in the environment and is useful for detecting objects in adverse weather conditions. Since the data generated by radar is usually small, the CAN FD communication method can meet the requirements. After receiving the heterogeneous data from the three sensors, the ADAS domain controller performs data fusion and, based on this, carries out path planning and final motion control decisions. The final control signals of the ADAS domain controller will be sent to the central gateway and then forwarded to the corresponding motion control ECUs, such as EPS, EM, and BCM, through the corresponding domain controller to achieve the corresponding vehicle longitudinal and lateral control, thus realizing the emergency avoidance of the AEB system to avoid collision. At the same time, the central gateway also sends the motion decision signals and warning signals of the ADAS domain controller to the IVI for display on the vehicle screen, making it convenient for the driver to view and warn. If an emergency occurs and the AEB system is triggered for emergency avoidance, the central gateway will also send the emergency signal to the TSP cloud through TBox to remind the OEMs and related departments to take emergency measures, ensuring the safety of passengers’ lives.

Based on the AEB system architecture diagram and communication signals, we can identify the assets in the AEB system. The threat analysis and risk assessment process in ISO/SAE 21434 defines a method for asset identification in the AEB system. In this example, assets in the AEB system are defined and classified into components, channels, messaging, and interfaces according to the CCMI communication model. The defined CCMI components are listed in Table 3, the defined CCMI channels are listed in Table 4, the defined CCMI messaging is listed in Table 5, and the defined CCMI interfaces are listed in Table 6.

**Figure 4:**
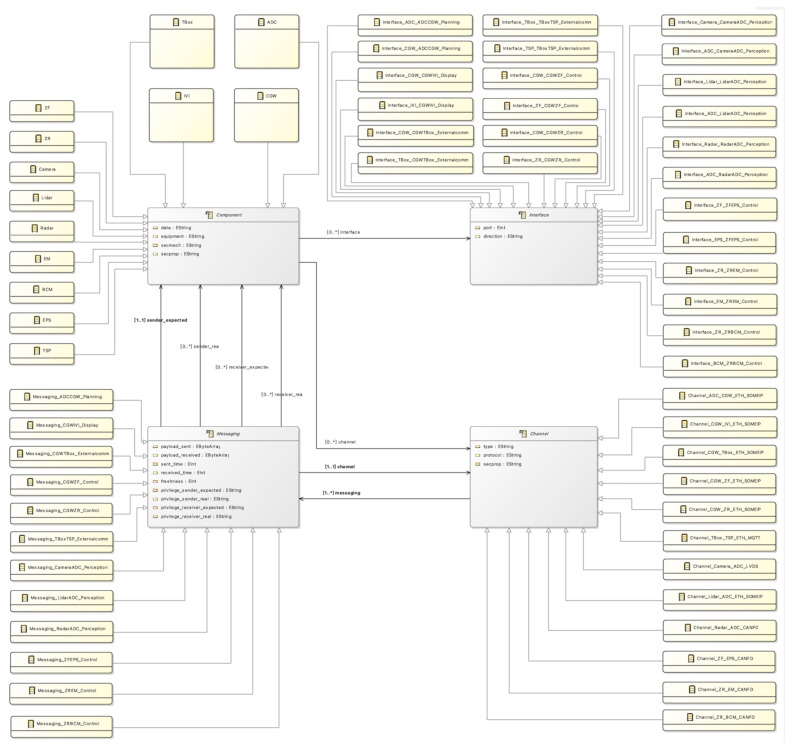
AEB class UML diagram in EMF.

Then, the assets identified in the CCMI communication model are represented using EMF in the Eclipse IDE. Firstly, the AEB system is defined using UML diagrams in EMF. Due to the size of the AEB system, it is not possible to fully display the details of the UML diagrams in EMF. Figure 4 shows the AEB Class UML Diagram, which represents the CCMI model and its instances in the system. After modeling the AEB system using UML in EMF, the ‘Generate Model Code’ function in the ‘.genmodel’ file of EMF is used to automatically generate Java model code using the CCMI communication model framework. Then, the Python-based translation engine is used to convert the Java file to Alloy file code using the mapping method provided in Table 2, resulting in an AEB system model defined in the Alloy language.

**Figure 5:**
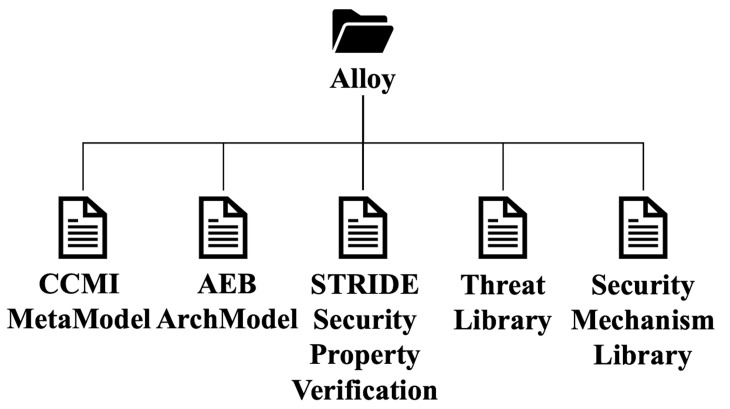
Alloy system model file structure.

The generated Alloy system model consists of five files, as shown in Figure 5. The file named ‘CCMI MetaModel’ contains the definitions of the four metamodels of the CCMI communication model, while the ‘AEB ArchModel’ file defines the instances of the CCMI model for the AEB system. These two files can be obtained by translating the Java model into EMF using the translation engine. This way, we can complete the AEB system modeling using the Alloy language. At the same time, the translation engine will also enumerate the instances of the AEB system’s CCMI model in the ‘STRIDE Security Property Verification’ file for later automated risk identification and traversal. The ‘Threat Library’ and ‘Security Mechanism Library’ files are the formal Threat Library and Security Mechanism Library established by the security experts using the Alloy language based on their previous security experience. By matching the elements in the CCMI communication model of the AEB system with the Threat Library and Security Mechanism Library in the ‘STRIDE Security Property Verification’ file, we can obtain the threats and risk points in the AEB system, as well as the corresponding security mechanisms and security requirements. This enables automated identification of system threats and risk points and provides corresponding security requirements.

**Figure 6:**
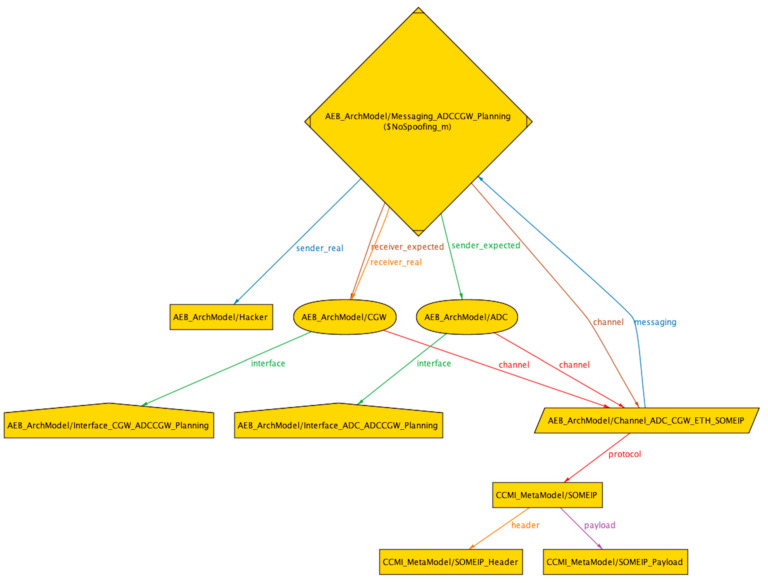
Counterexample of spoofing attack in AEB system.

**Figure 7:**
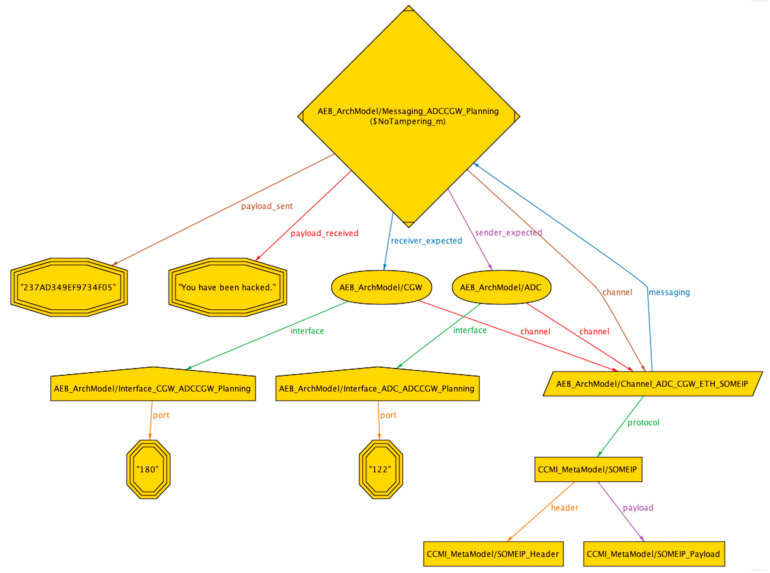
Counterexample of tampering attack in AEB system.

**Figure 8:**
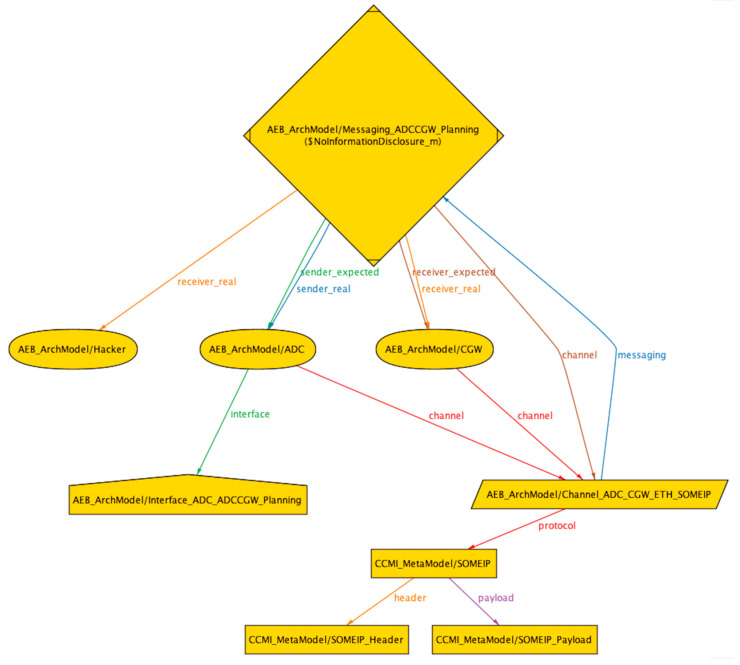
Counterexample of information disclosure attack in AEB system.

With the aforementioned file structure of the Alloy system, we can perform threat analysis and risk assessment based on the STRIDE threat model for assets in the system. We have established two libraries, namely the threat library and the security mechanism library, which match threats and security mechanisms with asset attributes in the libraries. In the ‘STRIDE Security Property Verification’ file, the asset attributes of AEB instances in the ‘AEB ArchModel’ file are traversed and matched with the threat and security mechanism libraries using Alloy’s ‘*asset*’ and ‘*check*’ syntax. If a counterexample is found during the search, indicating that the asset matches the threat and is at risk of attack, the output will show ‘*Counterexample found*’. If a security requirement from the security mechanism library is enabled and the threat can no longer be matched to the asset, it means that the security requirement is matched with the threat and thus provides an automated mechanism for matching asset threats and security mechanisms. In this article, we provide graphical representations of counterexamples matched to three typical threats, namely spoofing, tampering, and information disclosure. The algorithm for checking the spoofing threat is given in Formula (6), and the visualization of the counterexample is shown in Figure 6. The algorithm for checking the tampering threat is given in Formula (7), and the visualization of the counterexample is shown in Figure 7. The algorithm for checking the information disclosure threat is given in Formula (9), and the visualization of the counterexample is shown in Figure 8.

The AEB system, as a critical function in intelligent connected vehicles, involves multiple system components and has high system interdependence. Therefore, the threat analysis and risk assessment process for the AEB system is quite lengthy and complex. After conducting a comprehensive analysis of the AEB system, we obtained the final experimental results. To evaluate the superiority of the formalized and automated threat analysis and risk assessment method proposed in this paper for the CRMS system, we compared this method with the HEAVENS threat analysis and risk assessment method. The HEAVENS method provides a systematic approach and a complete evaluation process, allowing threat modeling and assessment to be applied throughout the entire software development cycle. Its structured approach enables rapid identification of security issues, improving the manageability and efficiency of system security. As a result, the HEAVENS method is recommended in international regulations for vehicle information security, such as SAE J3061, and has been widely used in threat analysis and risk assessment activities by major OEMs and their suppliers in the automotive industry. In addition, the HEAVENS method uses the STRIDE threat model in its threat analysis process, which is consistent with the threat analysis model in the CRMS framework proposed in this paper. This enables a direct comparison of threat detection rates and coverage rates of security requirements for the six types of threats, making the experimental results of this paper more convincing. Therefore, the HEAVENS method was chosen to be compared with the CRMS method proposed in this paper.

**Figure 9:**
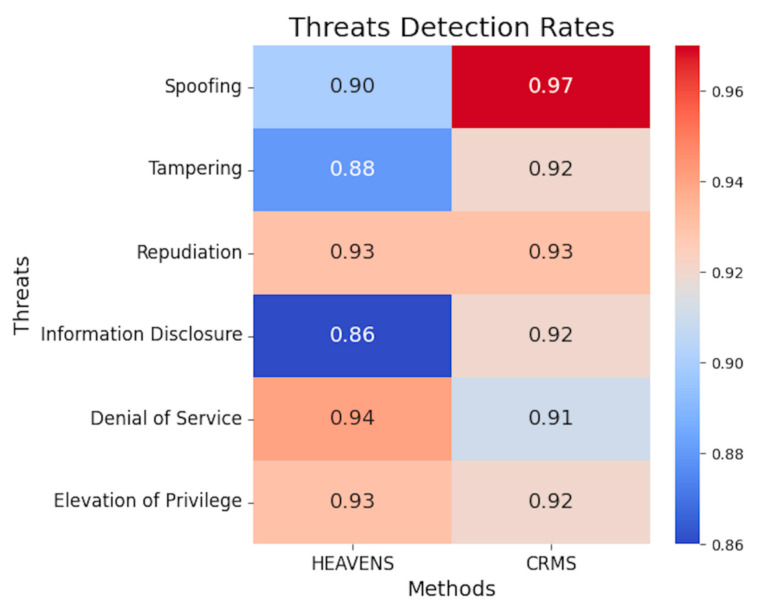
Threat detection rates of methods.

Firstly, the threat analysis and risk assessment processes require accurate identification of corresponding threats. Failure to identify threats in the system can lead to the existence of many vulnerabilities, thereby increasing the possibility of attacks on the system. Therefore, the first step is to evaluate whether the threat detection rates of the evaluation method can be improved. We used the two methods mentioned above to perform threat analysis and risk assessment on the AEB system and compared their results with known threats in the real system. The results of threat detection rates are shown in Figure 9. It can be seen that the threat detection rates of the proposed method are higher than the HEAVENS method in spoofing, tampering, and information disclosure, all exceeding 92%. The detection rates for spoofing threats reached 97%, which is a 7% improvement over the HEAVENS method. Since spoofing is a common threat in automotive systems, this improvement can help OEMs detect more spoofing threats, thereby effectively improving the overall cybersecurity of vehicles. Moreover, the detection rate for information disclosure threats increased from 86% to 92%, which is a 6% increase compared to the HEAVENS method. As another common threat in vehicle systems, identifying more confidential information leakage risks more accurately means that OEMs can block many malicious attacks on vehicles from the source, greatly reducing the attack surface of vehicles and the feasibility of other threats. However, the threat detection rates for denial of service and elevation of privilege are slightly lower than the HEAVENS method but still within an acceptable range.

**Figure 10:**
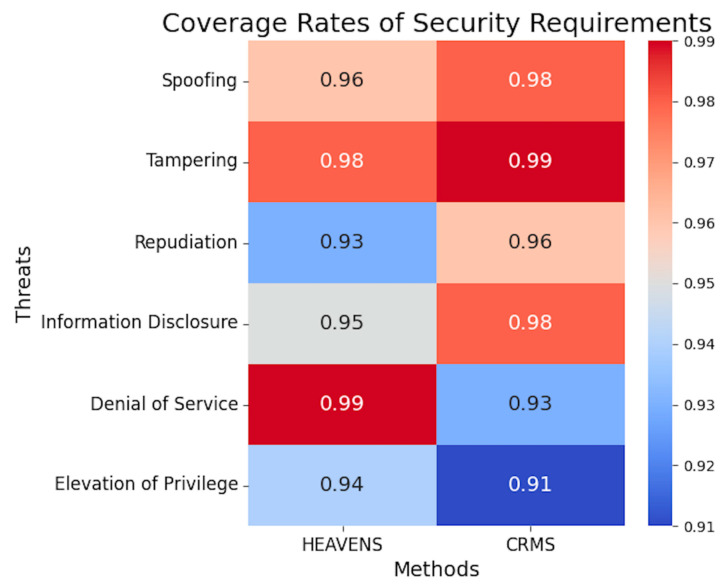
Coverage rates of security requirements of methods.

After the system detects threats and risks, it is necessary to provide corresponding security requirements to mitigate the identified threats and risks. If the system can detect threats and risks but cannot provide appropriate security requirements, or if the given security requirements cannot mitigate the identified threats and risks, then the system has not achieved the desired effect. Therefore, we evaluated the ability of the system to match security requirements to identified threats and risks using the coverage rates of security requirements. The results are shown in Figure 10. It can be seen that the coverage rates of security requirements of the method proposed in this paper are higher than those of the HEAVENS method in spoofing, tampering, repudiation, and information disclosure. Especially, the coverage rates of spoofing, tampering, and information disclosure are 98%, 99%, and 98%, respectively. We believe that security requirements with coverage rates like these are already suitable for practical applications. Spoofing, tampering, and information disclosure are three common threats in automotive systems. Improving the coverage rates of security requirements for these three threats is of great significance. This means that once the system successfully identifies these three threats, it can automatically match them with corresponding effective security measures, thus achieving a timely and effective defense against them. Additionally, accurately and correctly matching security measures for identified threats can effectively defend against almost all identified threats and risks at the conceptual stage, thereby avoiding product iteration caused by the discovery of new threats and risks in subsequent development and testing phases. This can greatly reduce OEMs’ costs of repetitive development in terms of both money and time.

**Figure 11:**
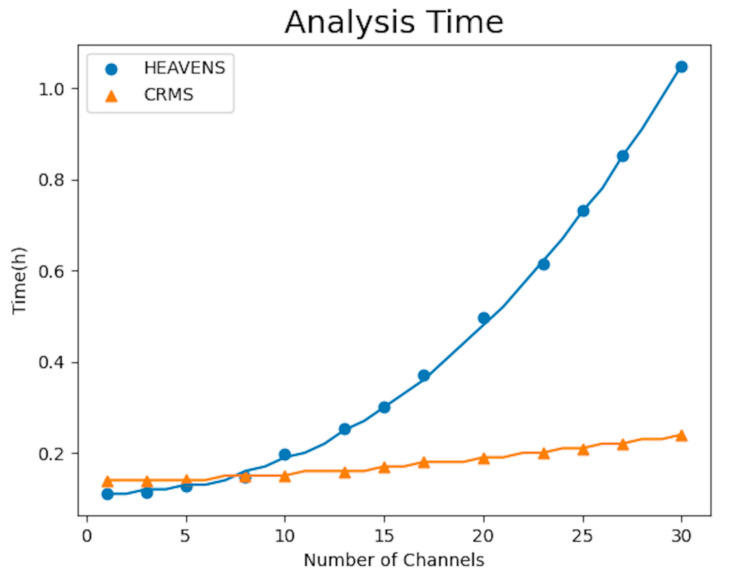
Analysis Time of Methods.

In addition, a significant advantage of the proposed method is its automation, which can help companies save time and money when conducting threat analysis and risk assessment on their systems. In order to assess the efficacy of the automated threat analysis and risk assessment approach proposed in this study, a comparative analysis was performed between the proposed method and the HEAVENS method for threat analysis and risk assessment in systems with varying numbers of channels. The time required for these analyses was used as the performance metric. The number of channels in a system can represent its complexity, and we fitted the experimental results to obtain the relationship between the analysis time and system complexity. The results are shown in Figure 11. As shown in Figure 11, when the system is relatively simple, the proposed method in this paper requires slightly more time than the HEAVENS method, mainly due to the need to use UML to model the system with CCMI. However, as the system complexity increases, at around 8 channels, the analysis time required by the proposed method is lower than that required by the HEAVENS method. Moreover, as the system complexity further increases, the growth curve of the analysis time required by the proposed method remains slow, while the HEAVENS method shows a significant increase in analysis time. Therefore, the proposed automated method can save more time compared to the HEAVENS method, and the efficiency advantage of the proposed method in this paper becomes more apparent. In conclusion, the proposed automated method in this paper has a more significant effect on reducing the time cost of threat analysis and risk assessment in large and complex systems, and this effect becomes more apparent as the system complexity increases.

## 6. Conclusions

In this paper, we have proposed a cybersecurity requirements management framework for the automotive domain. This method can help security experts transform their security expertise into formal language using the Alloy tool and establish a formal threat and security requirement library. Moreover, software development engineers can quickly model systems in UML form by using the EMF framework in this method. By using the CCMI communication framework proposed in this method, formal and informal models can be mapped, helping software development engineers obtain corresponding security requirements before developing code and assisting security experts in quickly and accurately identifying system risks in bulk. The case study in this paper demonstrated that our proposed approach outperforms the commonly used HEAVENS approach in terms of threat detection rates and coverage rates of security requirements. The proposed method exhibits higher rates of threat detection than the HEAVENS method across the categories of spoofing, tampering, and information disclosure, all surpassing 92%. In particular, the rate of detection for spoofing threats reached 97%. The comparison of the coverage rates of security requirements between the proposed method and the HEAVENS method indicates that the former outperforms the latter in the categories of spoofing, tampering, repudiation, and information disclosure. Notably, the coverage rates for spoofing, tampering, and information disclosure reach 98%, 99%, and 98%, respectively. Our method is more efficient in terms of threat analysis time for large-scale complex systems with more than 8 channels, and its effectiveness becomes more prominent with the increasing complexity of the system.

In the future, we plan to extend our framework to analyze larger and more complex automotive systems. Additionally, we will perform more adaptation work for the latest communication protocols, such as time-sensitive networking (TSN) and industry-specific standards like Diagnostics over Internet Protocol (DoIP), to make our architecture more comprehensive in adapting to the automotive industry. These identified threats and security requirements will be added to our formalized threat and security requirements library, making our proposed framework more comprehensive in identifying security risks and performing accurate matching in the system. We also plan to compare our method’s performance with other industry methods, such as SAHARA, to further validate the superiority of our approach. Moreover, we will continue to improve our method, making it more automated and efficient and adding functionality to generate risk reports for building an integrated system. In addition to identifying all the risks in the system and matching them with the corresponding security requirements using Alloy, we will also generate a comprehensive system risk assessment report. This report will provide detailed information on the identified risks, their potential impact, and recommendations for mitigating them. Furthermore, since the Alloy tool is open-source, the risk assessment report generation feature will be integrated into the tool as a plugin, which will enable users to quickly and easily generate risk assessment reports. This will enhance the usability and agility of the framework, making it an ideal choice for conducting system risk assessments in the automotive domain.

## Figures and Tables

**Table 1:** Advantages and disadvantages of relevant methods.

Category	Methods	Advantages/Disadvantages
Formula-based	EVITA	No complete evaluation process.
	HEAVENS	HEAVENS provides a detailed process.
	SHIELD	SHIELD enables the evaluation of multiple system configurations and the selection of those that meet established requirements.
	SGM	Easy to use. Difficulty in describing complex systems in detail.
	TVRA	Quantification. Long analysis time is required.
	SAHARA	Safety considered. Long analysis time is required.
	FMVEA	Failure modes are analyzed in terms of component quality attributes, while threat modes are used to analyze security attribute failures.
	CHASSIS	Too many subjective factors in the analysis method.
	ANP	ANP considered the relationship between failures and threats and the impact of propagation, which can help reduce the number of design iterations.
	SARA	SARA provides a framework for security experts to participate in the security process.
	SAM	SAM combines safety management and model-based system engineering through an abstract description of the principles of automotive security modeling. High learning costs.
	Bayesian Stackelberg Game	Can reduce the impact of advanced persistent threats. High learning costs.
Model-based	STRIDE	Be able to identify and analyze the threats in the system. Need to expand and adapt to the automotive field.
	PASTA	PASTA utilizes data flow diagrams at the application decomposition layer.
	GTS	High learning costs.
	UMLsec	Unable to perform detailed representations for complex, large systems.
	UML2Alloy	Need to expand and adapt to the automotive field.
	Attack Tree Analysis	Long analysis time is required.
	RISKEE	Long analysis time is required.
	STPA-Sec	Did not consider the network and system architecture.
	STPA-SafeSec	Did not consider the network and system architecture.

**Table 2:** Mapping rules between EMF elements and Alloy elements.

Rules	EMF Elements	Alloy Elements
Rule 1	EObject	Sig
Rule 2	EClass	Sig
Rule 3	EList	Set
Rule 4	EStructuralFeature	Sig
Rule 5	Void	Fun

**Table 3:** Identified component assets in AEB system.

Category	Assets	Description
Core Component	CGW	Central Gateway
Core Component	ADC	ADAS Domain Controller
Core Component	IVI	In-Vehicle Infotainment
Core Component	TBox	Telematics-Box
Domain Controller	ZF	Zone Front
Domain Controller	ZR	Zone Rear
Sensor	Camera	Camera
Sensor	Lidar	Lidar
Sensor	Radar	Radar
ECU	EM	Electric Motor
ECU	BCM	Brake Control Module
ECU	EPS	Electric Power Steering
Offcar	TSP	Telematics Service Provider

**Table 4:** Identified channel assets in AEB system.

Assets	Communication Objects	Channel Type
Channel_ADC_CGW_ETH_SOMEIP	ADC-CGW	Ethernet
Channel_CGW_IVI_ETH_SOMEIP	CGW-IVI	Ethernet
Channel_CGW_TBox_ETH_SOMEIP	CGW-TBox	Ethernet
Channel_CGW_ZF_ETH_SOMEIP	CGW-ZF	Ethernet
Channel_CGW_ZR_ETH_SOMEIP	CGW-ZR	Ethernet
Channel_TBox_TSP_ETH_MQTT	TBox-TSP	Ethernet
Channel_Camera_ADC_LVDS	Camera-ADC	LVDS
Channel_Lidar_ADC_ETH_SOMEIP	Lidar-ADC	Ethernet
Channel_Radar_ADC_CANFD	Radar-ADC	CAN FD
Channel_ZF_EPS_CANFD	ZF-EPS	CAN FD
Channel_ZR_EM_CANFD	ZR-EM	CAN FD
Channel_ZR_BCM_CANFD	ZR-BCM	CAN FD

**Table 5:** Identified messaging assets in AEB system.

Assets	Channel Type	Channel
Messaging_ADCCGW_Planning	Ethernet	Channel_ADC_CGW_ETH_SOMEIP
Messaging_CGWIVI_Display	Ethernet	Channel_CGW_IVI_ETH_SOMEIP
Messaging_CGWTBox_Externalcomm	Ethernet	Channel_CGW_TBox_ETH_SOMEIP
Messaging_CGWZF_Control	Ethernet	Channel_CGW_ZF_ETH_SOMEI
Messaging_CGWZR_Control	Ethernet	Channel_CGW_ZR_ETH_SOMEIP
Messaging_TBoxTSP_Externalcomm	Ethernet	Channel_TBox_TSP_ETH_MQTT
Messaging_CameraADC_Perception	LVDS	Channel_Camera_ADC_LVDS
Messaging_LidarADC_Perception	Ethernet	Channel_Lidar_ADC_ETH_SOMEIP
Messaging_RadarADC_Perception	CAN FD	Channel_Radar_ADC_CANFD
Messaging_ZFEPS_Control	CAN FD	Channel_ZF_EPS_CANFD
Messaging_ZREM_Control	CAN FD	Channel_ZR_EM_CANFD
Messaging_ZRBCM_Control	CAN FD	Channel_ZR_BCM_CANFD

**Table 6:** Identified interface assets in AEB system.

Assets	Component	Direction
Interface_ADC_ADCCGW_Planning	ADC	Output
Interface_CGW_ADCCGW_Planning	CGW	Input
Interface_CGW_CGWIVI_Display	CGW	Output
Interface_IVI_CGWIVI_Display	IVI	Input
Interface_CGW_CGWTBox_Externalcomm	CGW	Output
Interface_TBox_CGWTBox_Externalcomm	TBox	Input
Interface_TBox_TBoxTSP_Externalcomm	TBox	Output
Interface_TSP_TBoxTSP_Externalcomm	TSP	Input
Interface_CGW_CGWZF_Control	CGW	Output
Interface_ZF_CGWZF_Control	ZF	Input
Interface_CGW_CGWZR_Control	CGW	Output
Interface_ZR_CGWZR_Control	ZR	Input
Interface_Camera_CameraADC_Perception	Camera	Output
Interface_ADC_CameraADC_Perception	ADC	Input
Interface_Lidar_LidarADC_Perception	Lidar	Output
Interface_ADC_LidarADC_Perception	ADC	Input
Interface_Radar_RadarADC_Perception	Radar	Output
Interface_ADC_RadarADC_Perception	ADC	Input
Interface_ZF_ZFEPS_Control	ZF	Output
Interface_EPS_ZFEPS_Control	EPS	Input
Interface_ZR_ZREM_Control	ZR	Output
Interface_EM_ZREM_Control	EM	Input
Interface_ZR_ZRBCM_Control	ZR	Output
Interface_BCM_ZRBCM_Control	BCM	Input

## Data Availability

Not applicable.
